# Interpretable and generalizable deep learning model for preoperative assessment of microvascular invasion and outcome in hepatocellular carcinoma based on MRI: a multicenter study

**DOI:** 10.1186/s13244-025-02035-0

**Published:** 2025-07-03

**Authors:** Xue Dong, Xibin Jia, Wei Zhang, Jingxuan Zhang, Hui Xu, Lixue Xu, Caili Ma, Hongjie Hu, Jiawen Luo, Jingfeng Zhang, Zhenchang Wang, Wenbin Ji, Dawei Yang, Zhenghan Yang

**Affiliations:** 1 Department of Radiology, Beijing Friendship Hospital, Capital Medical University, Beijing, China; 2 College of Computer Science, Beijing University of Technology, Beijing, China; 3 Department of Radiology, Beijing Longfu Hospital, Beijing, China; 4 Department of Radiology, Sir Run Run Shaw Hospital, Zhejiang University School of Medicine, Hangzhou, China; 5 Department of Radiology, The Second Hospital of Dalian Medical University, Dalian, China; 6 Department of Radiology, Ningbo No. 2 Hospital, Ningbo, China; 7 Department of Radiology, Taizhou Hospital of Zhejiang Province Affiliated to Wenzhou Medical University, Linhai, China; 8 Key Laboratory of Evidence-based Radiology of Taizhou, Linhai, China

**Keywords:** Hepatocellular carcinoma, Microvascular invasion, Deep learning model, Generalization, Interpretability

## Abstract

**Objectives:**

This study aimed to develop an interpretable, domain-generalizable deep learning model for microvascular invasion (MVI) assessment in hepatocellular carcinoma (HCC).

**Methods:**

Utilizing a retrospective dataset of 546 HCC patients from five centers, we developed and validated a clinical-radiological model and deep learning models aimed at MVI prediction. The models were developed on a dataset of 263 cases consisting of data from three centers, internally validated on a set of 66 patients, and externally tested on two independent sets. An adversarial network-based deep learning (AD-DL) model was developed to learn domain-invariant features from multiple centers within the training set. The area under the receiver operating characteristic curve (AUC) was calculated using pathological MVI status. With the best-performed model, early recurrence-free survival (ERFS) stratification was validated on the external test set by the log-rank test, and the differentially expressed genes (DEGs) associated with MVI status were tested on the RNA sequencing analysis of the Cancer Imaging Archive.

**Results:**

The AD-DL model demonstrated the highest diagnostic performance and generalizability with an AUC of 0.793 in the internal test set, 0.801 in external test set 1, and 0.773 in external test set 2. The model’s prediction of MVI status also demonstrated a significant correlation with ERFS (*p* = 0.048). DEGs associated with MVI status were primarily enriched in the metabolic processes and the Wnt signaling pathway, and the epithelial-mesenchymal transition process.

**Conclusions:**

The AD-DL model allows preoperative MVI prediction and ERFS stratification in HCC patients, which has a good generalizability and biological interpretability.

**Critical relevance statement:**

The adversarial network-based deep learning model predicts MVI status well in HCC patients and demonstrates good generalizability. By integrating bioinformatics analysis of the model’s predictions, it achieves biological interpretability, facilitating its clinical translation.

**Key Points:**

- Current MVI assessment models for HCC lack interpretability and generalizability.
- The adversarial network-based model's performance surpassed clinical radiology and squeeze-and-excitation network-based models.
- Biological function analysis was employed to enhance the interpretability and clinical translatability of the adversarial network-based model.

**Graphical Abstract:**

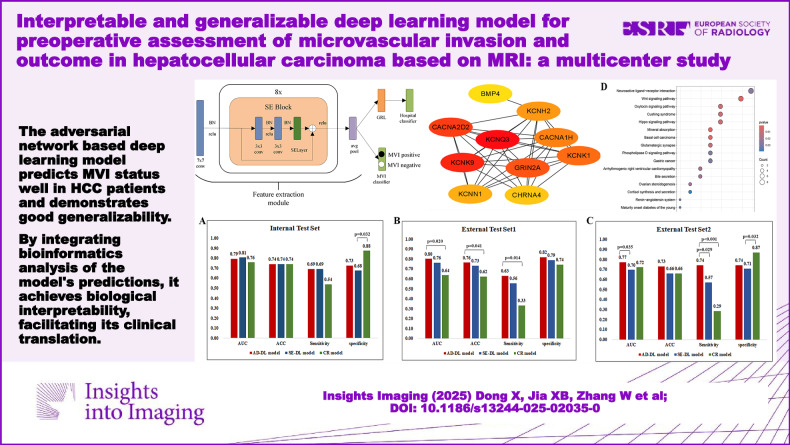

## Introduction

Hepatocellular carcinoma (HCC) ranks as the third leading cause of cancer-related mortality globally [1]. Curative treatment options for early or partial intermediate HCC include liver resection and liver transplantation; however, the efficacy of these surgical interventions remains limited due to several factors, such as high rates of early recurrence, vascular invasion resulting in intrahepatic dissemination, and occult metastases [2]. The 5-year recurrence rate following liver resection can reach up to 70% [3].

Microvascular invasion (MVI) is a critical pathological feature in the progression of HCC [4, 5]. MVI is defined as the presence of tumor cell nests within the lumina of blood vessels lined by endothelial cells, primarily involving branches of the periportal vein, including vessels within the tumor capsule [6, 7]. It serves as an independent prognostic factor for HCC, with substantial evidence indicating that a positive MVI status is associated with more aggressive tumor behavior, higher early recurrence rates, and reduced overall survival [8–10]. Preoperative identification of MVI is essential for optimizing treatment strategies in HCC. If a high risk of MVI is anticipated preoperatively, a wider resection margin may be warranted to mitigate recurrence risk. In HCC tumors ≤ 3 cm, liver resection is generally favored over radiofrequency ablation [11–13]. Thus, accurate pre-treatment identification of MVI status holds significant clinical value.

However, the diagnosis of MVI in HCC relies exclusively on pathological evaluation following surgical resection. Liver contrast-enhanced MRI, with its advantages of high soft tissue contrast and multiparametric, multidirectional imaging, has been widely utilized for the diagnosis, staging, and therapeutic assessment of HCC [14, 15]. MRI-based deep learning algorithms have the capability to extract and quantify subtle radiological features that are imperceptible to the human eye, thereby enabling the development of targeted models. These models provide a non-invasive tool for diagnosing and evaluating various diseases, including HCC [16]. While numerous MRI-based deep learning models for MVI prediction have been proposed, there is considerable variation across studies, most of which are limited to single-center or dual-center cohorts [17–20], with insufficient evidence regarding their generalizability to multi-center settings. Traditional deep learning models are typically trained on data from a single institution; however, variations in MRI sequence protocols and acquisition parameters across different hospitals often introduce data biases, resulting in limited generalizability when applied in a multicenter setting. To address these challenges, domain generalization [21, 22] has been adopted. Domain generalization training methods based on multi-source domains have been shown to effectively improve the model’s generalizability to previously unseen target domains [23]. However, this approach has not yet been explored in the context of MVI evaluation in HCC patients. Therefore, we explored a domain generalization training approach based on multicenter data, further referencing a promising adversarial network strategy from natural image analysis for achieving domain generalization [24–26].

Therefore, this study aimed to develop a domain-generalized deep learning model using adversarial training to assess MVI in HCC from preoperative MRI, and to validate across multiple test sets. Additionally, we sought to identify differentially expressed genes (DEGs) associated with the predicted MVI status to elucidate the biological functions, thereby enhancing the model’s interpretability.

## Materials and methods

### Patients

This study was approved by the ethics review committees of all participating tertiary referral centers, including Beijing Friendship Hospital, Capital Medical University (Beijing center); Sir Run Run Shaw Hospital, School of Medicine, Zhejiang University (Hangzhou center); Ningbo Huamei Hospital, University of Chinese Academy of Sciences (Ningbo center); Taizhou Hospital, Zhejiang University (Taizhou center); and the Second Affiliated Hospital of Dalian Medical University (Dalian center). Informed consents from patients were waived.

**Fig. 1:**
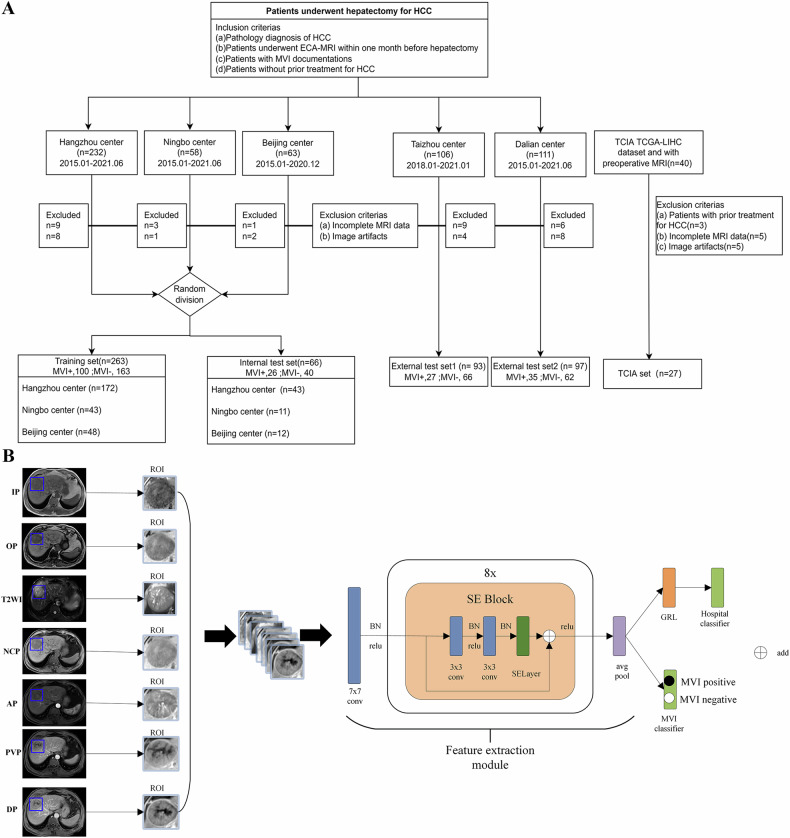
Patient selection and study design. **A** Flow diagram of the patient selection process from five medical centers and the TCIA dataset, and (**B**) the overall architecture of the deep learning network. AP, the arterial phase; BN, batch normalization; DP, delayed phase; ECA, extracellular contrast medium; GRL, gradient-reversal layer; HCC, hepatocellular carcinoma; IP, in-phase; MVI, microvascular invasion; MRI, magnetic resonance imaging; NCP, non-contrast phase; OP, opposed-phase; PVP, portal venous phase; ROI, region of interest; SE, squeeze-and-excitation; TCGA-LIHC, the cancer genome atlas liver hepatocellular carcinoma; TCIA, the cancer image archive; T2WI, T2-weighted imaging

We conducted a retrospective study involving the continuous collection of data from 610 patients with HCC who underwent liver resection across five medical centers from January 2015 to January 2021, as well as from the Cancer Imaging Archive (TCIA) (Fig. 1A). Details are provided in the Supplementary Appendixes 1 and 3.

To construct the model, patients from the Beijing center, Hangzhou center, and Ningbo center were randomly assigned to the training set and internal test set in a 4:1 ratio. The model was further validated using external test sets from the Taizhou center and the Dalian center. The results cohort from the Taizhou center was utilized to evaluate the prognostic risk stratification capabilities of the proposed MVI prediction model, while the TCIA cohort was employed to explore the biological mechanisms associated with MVI.

### Clinical characteristics and outcomes

Clinical data were retrospectively collected from electronic medical records. MVI is defined as the presence of tumor cell nests in the portal veins, hepatic veins, or vessels lined with endothelium within the tumor capsule, as observed under a microscope [6, 7]. Early recurrence-free survival (ERFS) was defined as the time interval from surgery to the occurrence of intrahepatic recurrence or extrahepatic metastasis within two years. Details are provided in the supplementary appendix 1.

### MRI scan and evaluation of MRI features

All patients underwent MRI scans using an 8-channel abdominal coil. The MRI protocol was in accordance with the standards recommended by the American Association for the Study of Liver Diseases (AASLD) [27]. A summary of the MRI scan is provided in Table S1 and supplementary appendix 1.

The MRI feature evaluation was based on prior studies and the definitions provided in the 2018 edition of the Liver Imaging Reporting and Data System (LI-RADS) [28–32]. The evaluation methods and MRI feature details are provided in the supplementary appendix 1.

### Clinical–radiological model

Clinical and MRI features with a *p* value < 0.05 in the univariate logistic regression analysis were included in the multivariate logistic regression model using a stepwise selection approach. Model simplification was performed based on the Akaike information criterion (AIC), and the model with the lowest AIC value was selected to generate the final clinical-radiological (CR) model.

### Deep learning model

The tumor regions were manually delineated on the maximum cross-sectional tumor areas of seven sequences: in-phase (IP) and opposed-phase (OP), T2-weighted imaging (T2WI), and non-contrast phase (NCP), the arterial phase (AP), portal venous phase (PVP), and delayed phase (DP). Through different channel combinations within the convolutional neural network, experiments were conducted using various sequence combinations for further analysis.

We design an adversarial network to learn domain-invariant features from multiple hospitals in the training set. The main architecture of the deep learning network (Fig. 1B) can be divided into four main parts, namely, the feature extraction module, the MVI classifier, the hospital classifier and the gradient-reversal layer (GRL). We employ a gradient-weighted class-activation mapping approach to elucidate the important regions of the image used for prediction. A detailed description of the deep learning model and training process can be found in the Supplementary Appendix 2.

### Statistical analysis

Univariate and multivariate logistic regression analyses were performed to identify variables independently associated with MVI status. Cohen’s κ statistic and the intraclass correlation coefficient were used to assess inter-observer consistency. The area under the curve (AUC) of the receiver operating characteristic (ROC) curve was calculated to evaluate the predictive power of both the clinical radiological and domain-generalized deep learning models. The DeLong test was used to compare the predictive performance of models based on AUC, while sensitivity, specificity, and accuracy (ACC) were compared using the *Z* test. The optimal model was determined based on results from the internal test set Kaplan–Meier survival curves were generated and compared with the log-rank test. All statistical analyses were conducted using R software (version 4.2.0, http://www.R-project.org) and SPSS 25.0 (version 25.0, https://www.ibm.com). A two-tailed *p* value < 0.05 was considered statistically significant. Further details on RNA sequencing analysis from the TCIA dataset and the association between these data and imaging features are available in the Supplementary Appendix 3.

## Results

### Clinical characteristics and radiologic features

Among the 610 patients who underwent surgery for HCC, 33 patients were excluded due to incomplete MRI data, 28 patients were excluded due to severe image artifacts, and 3 patients were excluded because they had received prior HCC treatment before MRI examination. Ultimately, 546 patients were included in the analysis. Detailed clinical and radiologic features for each group are provided in Tables 1 and  S2, with the exception of some missing patient data for the TCIA group. There were no statistically significant differences in clinical and radiologic features between the training and internal test groups (*p* > 0.05). Histological/Pathological results indicated that the MVI status was balanced across the training set, internal test set, external test set 1, and external test set 2 (*p* = 0.434) (Table S2).

**Table 1:** Baseline patient characteristics

Characteristic	Training set (*n* = 263)	Internal test set (*n* = 66)	External test set 1 (*n* = 93)	External test set 2 (*n* = 97)	TCIA set (*n* = 27)
Patient demographics					
Age (y)^*^	60 [51–67]	61 [52–65]	60 [55–67]	61 [56–69]	64 [50–69]
Sex (male)	211 (80.2)	56 (84.8)	71 (76.3)	75 (77.3)	17 (63.0)
HBV infection	217 (82.5)	52 (78.8)	88 (94.6)	76 (78.4)	NA
BCLC stage (0 or A)	241 (91.6)	64 (97)	77 (82.8)	92 (94.8)	NA
Child–Pugh grade (A)	202 (76.8)	45 (68.2)	77 (82.8)	50 (51.5)	NA
ALT (U/L)^*^	30 [20–51]	35 [23–55]	26.5 [19–37.5]	73 [35.5–140]	NA
AST (U/L)^*^	33 [25–48]	33 [25–54]	34 [26–45]	38 [26.8–107]	NA
ALB (g/L)^*^	40.8 [36.4–43.7]	41.1 [36.9–43.4]	41.2 [38–44]	33.0 [36.0–42.0]	NA
PT (s)^*^	13.2 [12.7–14.0]	13.6 [12.8–14.1]	14 [13.3–14.6]	13.6 [13.1–14.5]	NA
AFP (ng/mL)^*^	18.2 [3.9–277.2]	18.4 [4.7–98.5]	18.9 [4.9–313.8]	21.2 [3.5–216.2]	NA
Radiologic feature					
Maximum tumor diameter (cm)*	3.8 [2.5–5.7]	3.4 [2.1–5.4]	3.1 [2.0–4.6]	3.2 [2.0–5.9]	6.3 [3.9–9.2]
Tumer number (solitary)	242 (92)	63 (95.5)	85 (91.4)	89 (91.8)	27 (100)
Hypo-intensity on T1WI (present)	227 (86.3)	58 (87.9)	82 (88.2)	81 (83.5)	24 (88.9)
Blood products in mass (present)	67 (25.5)	13 (19.7)	16 (17.2)	27 (27.8)	10 (37.0)
Rim APHE (present)	12 (4.6)	2 (3)	3 (3.2)	5 (5.2)	2 (7.4)
Intratumor vascularity (present)	132 (50.2)	29 (43.9)	46 (49.5)	35 (36.1)	17 (63.0)
Intratumor necrosis (present)	54 (20.5)	11 (16.7)	11 (11.8)	14 (14.4)	3 (11.1)
Enhancing “capsule“ (present)	240 (91.3)	58 (87.9)	82 (88.2)	85 (87.6)	25 (92.6)
Incomplete “capsule” (present)	210 (79.8)	59 (89.4)	76 (81.7)	70 (72.2)	24 (88.9)
Nonperipheral washout (present)	224 (85.2)	53 (80.3)	72 (77.4)	73 (75.3)	22 (81.5)
Nonsmooth tumor margin (present)	181 (68.8)	46 (69.7)	61 (65.6)	63 (64.9)	22 (81.5)
Peritumoral mild-to-moderate T2 hyperintensity (present)	44 (16.7)	6 (9.1)	10 (10.8)	13 (13.4)	5 (18.5)
Peritumoral PVP hypoenhancement (present)	55 (20.9)	10 (15.2)	14 (15.1)	10 (10.3)	5 (18.5)
Peritumoral AP hyperenhancement (present)	77 (29.3)	19 (28.8)	26 (28)	18 (18.6)	5 (18.5)
MVI (present)	100 (38.0)	26 (39.4)	27 (29.0)	35 (36.1)	NA

Unless otherwise indicated, data are numbers of patients, and data in parentheses are percentages

*AFP* alpha-fetoprotein, *ALB* plasma albumin, *ALT* alanine aminotransferase, *AP* arterial phase, *APHE* arterial phase hyperenhancement, *AST* aspartate aminotransferase, *BCLC* Barcelona Clinic Liver Cancer, *HBV* hepatitis B virus, *IQR* interquartile range, *MVI* microvascular invasion, *NA* not applicable, *PT* prothrombin time, *PVP* portal venous phase, *TCIA* the cancer imaging archive

^*^ Data are medians, with interquartile ranges (IQRs) in parentheses

### Clinical–radiological model

The interobserver agreements for the MRI features were good to excellent (Cohen’s κ = 0.715–0.943) (Table S3). In the training set, univariate logistic regression analysis revealed that patients with positive MVI status were more likely to have an alpha-fetoprotein (AFP) level greater than 200 ng/mL (odds ratio [OR] = 1.98, *p* = 0.01) and a tumor diameter greater than 5 cm (OR = 1.92, *p* = 0.01). Compared to HCC lesions without MVI, those with MVI were more likely to exhibit hypo-intensity on T1WI (OR = 2.39, *p* = 0.04), intratumoral vascularity (OR = 1.77, *p* = 0.03), peritumoral PVP hypoenhancement (OR = 1.96, *p* = 0.03), and peritumoral AP hyperenhancement (OR = 3.35, *p* < 0.01). Multivariate logistic regression analysis identified peritumoral AP hyperenhancement(OR = 3.09, *p* < 0.01) as an independent predictor of MVI. No significant differences were observed for other clinical and radiologic features. Detailed results are provided in Table 2.

**Table 2:** Logistic regression analysis of variables for their association with MVI in patients in the training set

Characteristic	Univariable analysis		Multivariable analysis	
	OR (95% CI)	*p* value	OR (95% CI)	*p* value
Age (> 50 years)	0.71 (0.4–1.27)	0.25	NA	NA
Sex (male)	0.66 (0.36, 1.21)	0.18	NA	NA
HBV infection	1.5 (0.76–2.98)	0.25	NA	NA
BCLC stage (B or C)	1.14 (0.47–2.78)	0.77	NA	NA
Child–Pugh grade (B)	1.08 (0.6, 1.93)	0.81	NA	NA
ALT level (> 50 U/L)	1.05 (0.59–1.85)	0.88	NA	NA
AST level (> 40 U/L)	1.24 (0.73–2.09)	0.43	NA	NA
ALB level (> 40 g/L)	1.29 (0.78–2.13)	0.32	NA	NA
PT level (> 13 s)	0.87 (0.52–1.43)	0.58	NA	NA
AFP level (> 200 ng/mL)	1.98 (1.14–3.44)	0.01	1.78 (0.99, 3.17)	0.05
Maximum tumor diameter (> 5 cm)	1.92 (1.14–3.24)	0.01	NA	NA
Tumer number (solitary)	0.8 (0.31–2.06)	0.64	NA	NA
Hypo-intensity on T1WI (present)	2.39 (1.04–5.47)	0.04	NA	NA
Blood products in mass (present)	1.05 (0.59–1.85)	0.88	NA	NA
Rim APHE (present)	1.67 (0.52–5.33)	0.39	NA	NA
Intratumor vascularity (present)	1.77 (1.07–2.94)	0.03	1.50 (0.88, 2.55)	0.13
Intratumor necrosis (present)	0.95 (0.51–1.76)	0.87	NA	NA
Enhancing capsule (present)	0.64 (0.27–1.52)	0.31	NA	NA
Incomplete “capsule” (present)	1.72 (0.89–3.33)	0.11	NA	NA
Nonperipheral washout (present)	1.11 (0.55–2.26)	0.77	NA	NA
Nonsmooth tumor margin (present)	1.61 (0.93–2.81)	0.09	NA	NA
Peritumoral mild-to-moderate T2 hyperintensity (present)	1.62 (0.84–3.11)	0.15	NA	NA
Peritumoral PVP hypoenhancement (present)	1.96 (1.07–3.57)	0.03	NA	NA
peritumoral AP hyperenhancement (present)	3.35 (1.93–5.82)	< 0.01	3.09 (1.77, 5.45)	< 0.01

*AFP* alpha-fetoprotein, *ALB* plasma albumin, *ALT* alanine aminotransferase, *AP* arterial phase, *APHE* arterial phase hyperenhancement, *AST* aspartate aminotransferase, *BCLC* Barcelona Clinic Liver Cancer, *CI* confidence interval, *HBV* hepatitis B virus, *NA* not applicable, *OR* odds ratio, *PT* prothrombin time, *PVP* portal venous phase

**Fig. 2:**
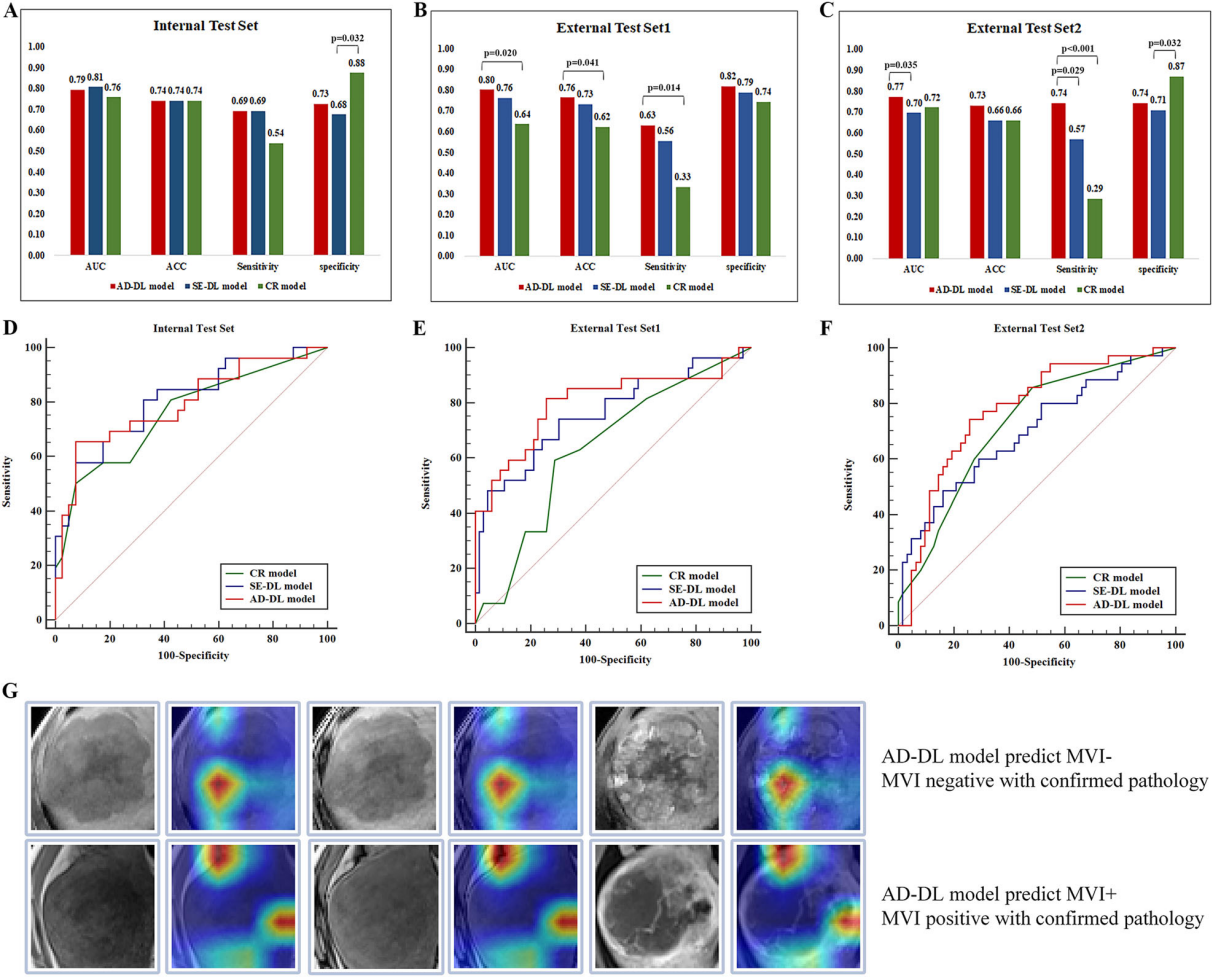
**A**–**C** The performance of the AD-DL model, the SE-DL model, and the CR model based on IP, OP, and AP was presented using bar charts, with results reported for the internal test set and external test sets 1 and 2. The DeLong test was used to compare the AUC, while the *Z* test was applied to assess sensitivity, specificity, and ACC. **D**–**F** The ROCs of the three models based on IP, OP, and AP in the internal test set, external test sets 1 and 2, respectively. **G** Two representative examples of MRI images and corresponding model predictions, Grad-CAM, which can elucidate the important regions of the image used for prediction. In the first case, the model predicted MVI to be negative, which was confirmed by pathology, and the Grad-CAM method identified and highlighted important regions within the tumor center on the MRI image. In the second case, the model predicted MVI to be positive, also confirmed by pathology, with the Grad-CAM method highlighting significant regions at the tumor margin. ACC, accuracy; AD-DL, adversarial network-based deep learning; AP, the arterial phase; AUC, area under the curve; CR, clinical radiology; IP, in-phase; MRI, magnetic resonance imaging; MVI, microvascular invasion; OP, opposed-phase; ROC, receiver operating characteristic. SE-DL, non-adversarial SENet-based deep learning

A CR model was developed using the AIC, incorporating three features: AFP level, intratumoral vascularity, and peritumoral AP hyperenhancement. The model’s cut-off value was 0.4595. The AUC for the internal test set was 0.759 (95% confidence interval [CI]: 0.638–0.856). For external test sets 1 and 2, the AUCs were 0.636 (95% CI: 0.530–0.733) and 0.721 (95% CI: 0.621–0.808), respectively. A detailed description of the model’s performance can be found in Table 3 and Fig. 2A–F.

**Table 3:** Diagnostic performance of models

Model and metric	CR	*SE-DL	*AD-DL	^a^*p* value	^b^*p* value	^c^*p* value
Internal test set (*n* = 66)						
Sensitivity	0.538	0.692	0.692	0.40	0.40	1.00
Specificity	0.875	0.675	0.725	0.032	0.09	0.63
ACC	0.742	0.742	0.742	1.00	1.00	1.00
AUC (95% CI)	0.759 (0.638–0.856)	0.807 (0.691–0.894)	0.793 (0.676–0.883)	0.546	0.667	0.680
External test1 set 1 (*n* = 92)						
Sensitivity	0.333	0.556	0.630	0.054	0.014	0.58
Specificity	0.742	0.788	0.818	0.42	0.21	0.66
ACC	0.623	0.731	0.763	0.12	0.041	0.62
AUC (95% CI)	0.636 (0.530–0.733)	0.762 (0.662–0.844)	0.801 (0.706–0.877)	0.094	0.020	0.239
External test set 2 (*n* = 97)						
Sensitivity	0.286	0.571	0.743	0.029	< 0.001	0.14
Specificity	0.871	0.710	0.742	0.032	0.064	0.84
ACC	0.660	0.660	0.732	1.00	0.35	0.35
AUC (95% CI)	0.721 (0.621–0.808)	0.696 (0.594–0.785)	0.773 (0.677– 0.852)	0.723	0.46	0.035

^*^ Model was trained with IP, OP, and AP sequences

*AD-DL* adversarial network-based deep learning, *AUC* area under the curve, *CI* confidence interval, *CR* clinical radiology, *SE-DL* non-adversarial SENet-based deep learning

^a^ Comparison between the CR and squeeze-and-excitation-deep learning (SE-DL) models

^b^ Comparison between the CR and AD-DL models

^c^ Comparison between the SE-DL and AD-DL models

### Deep learning model

In external test set 1, the application of the adversarial network-based deep learning (AD-DL) model resulted in a higher AUC in 66 out of 127 sequence combinations compared to the SENet-based deep learning (SE-DL) model. Similarly, in external test set 2, the AD-DL model resulted in an AUC improvement in 70 out of 127 sequence combinations compared to the SE-DL model. Across both external test sets, 38 out of 127 sequence combinations showed consistent AUC improvements following the AD-DL model. The diagnostic performance of the SE-DL and AD-DL models for sequence combinations with AUC > 0.7 is detailed in Table S4. The above experiments indicate that the optimal sequence combination was IP, OP, and AP, with a prediction threshold of 0.4843 for the DL model.

For the SE-DL model trained with IP, OP and AP sequence combination, the AUC was 0.807 (95% CI: 0.691–0.894) in the internal test set, 0.762 (95% CI: 0.662–0.844) in external tets set 1, and 0.696 (95% CI: 0.594–0.785) in external test set 2.

With the introduction of adversarial network, the AD-DL model using the same sequence combination demonstrated enhanced performance, achieving an AUC of 0.793 (95% CI: 0.676–0.883) in the internal test set, 0.801 (95% CI: 0.706–0.877) in external test set 1, and 0.773 (95% CI: 0.677–0.852) in external test set 2. A comprehensive summary of the SE-DL and AD-DL models’ diagnostic performance is presented in Table 3 and Fig. 2A–F.

### Model comparison

In external test set 1, the AD-DL model trained with IP, OP, and AP sequence combination outperformed the SE-DL model and CR model across all diagnostic performance metrics, with AUC, ACC, and sensitivity significantly higher than those of the CR model (*p* = 0.020, 0.041, 0.014, respectively). In external test test 2, the AD-DL model also demonstrated higher values for AUC, ACC, and sensitivity compared to the SE-DL and CR models, with AUC significantly outperforming the SE-DL model (*p* = 0.035), and sensitivity significantly higher than both the SE-DL and CR models (*p* = 0.029, < 0.001, respectively), while specificity was comparable to that of the other two models. Table 3 and Fig. 2A–F present a detailed comparison of the performance metrics for the CR, SE-DL, and AD-DL models.

Figure 2G presents two cases illustrating the prediction of MVI using the AD-DL model trained with IP, OP and AP sequences.

### Clinical outcomes and biologic functions associated with MVI status

**Fig. 3:**
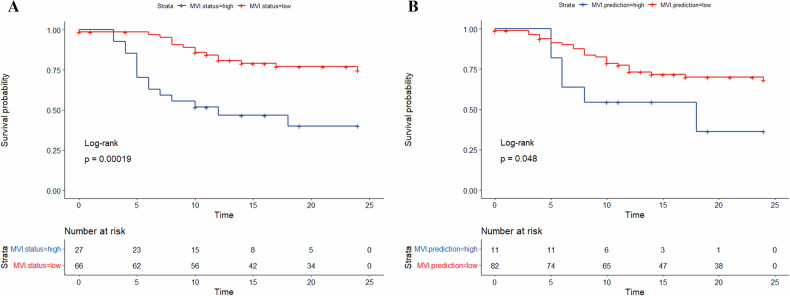
ERFS curves according to histological and AD-DL model based on IP, OP, and AP predicted MVI status. **A** ERFS curve scaled histological MVI status. **B** ERFS curves scaled AD-DL model based on IP, OP, and AP predicted MVI status. AD-DL, adversarial network-based deep learning; AP, the arterial phase; ERFS, early recurrence-free survival; IP, in-phase; MVI, microvascular invasion; OP, opposed-phase

Among the 93 patients in the Taizhou Center, the median follow-up period was 12 months, during which 27 patients experienced early recurrence of HCC. The mean ERFS for patients with MVI-positive HCC was 14.1 months (95% CI: 10.6–17.5 months), whereas for patients with MVI-negative HCC, the mean ERFS was significantly longer, at 20.7 months (95% CI: 19.1–22.3 months) (log-rank test, *p* < 0.05) (Fig. 3A). Notably, the AD-DL model, based on the optimal sequence combination of IP, OP, and AP, demonstrated a significant correlation with ERFS (*p* = 0.048) (Fig. 3B).

In the TCIA dataset, 198 DEGs associated with the MVI status predicted by the AD-DL model, based on the optimal sequence combination of IP, OP, and AP, were identified.

**Fig. 4:**
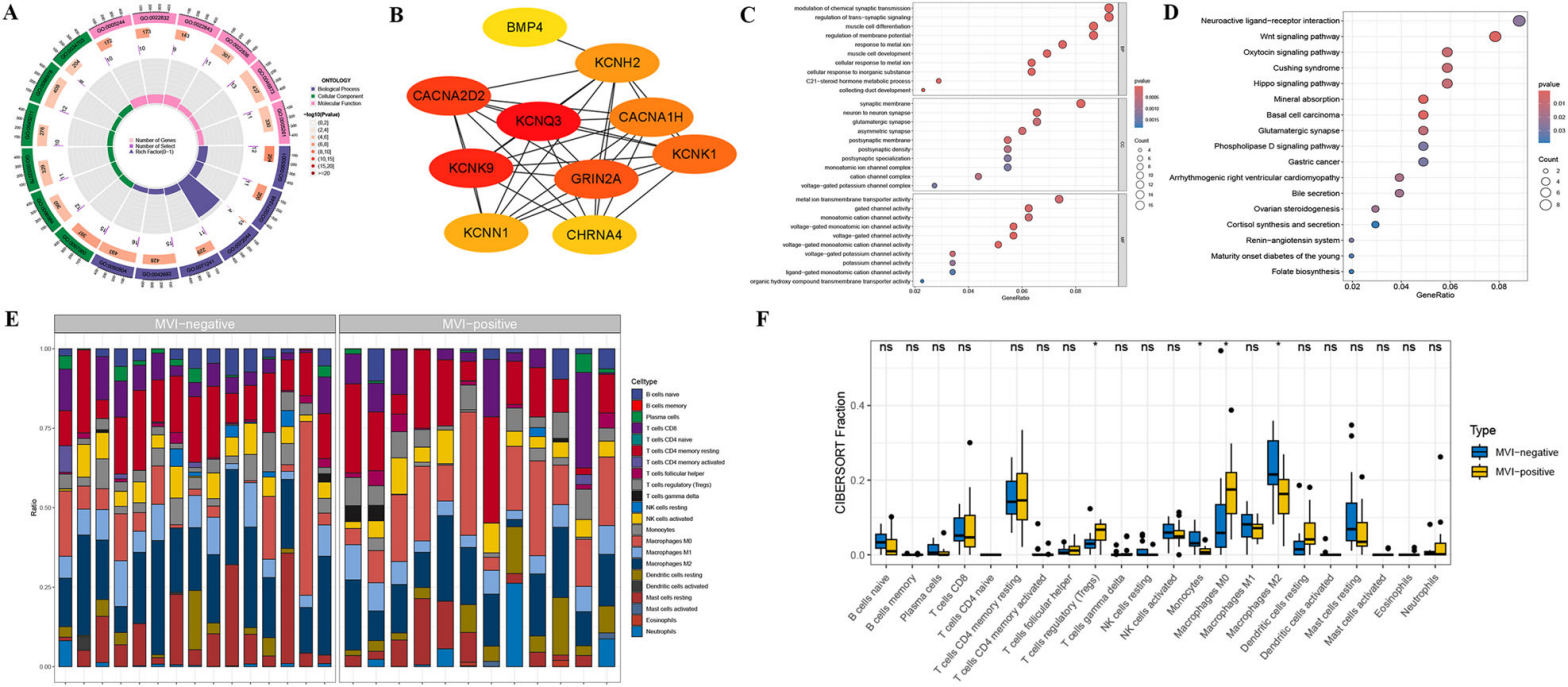
DEGs and measurement of immune cell tumor infiltration associated with the MVI status predicted by the AD-DL model, based on the optimal sequence combination of IP, OP, and AP, using RNA sequencing data from TCIA (*n* = 27). Functional enrichment analysis of DEGs was performed. **A** Circos Plot: indicating the relationship between genes and GO terms. **B** PPI network shows the top 10 hub genes identified with a maximal clique centrality algorithm in Cytoscape software. Bubble plots show results of the analysis when using (**C**) GO or (**D**) KEGG. *GeneRatio* means the ratio of genes in this pathway to all genes. *Count* means the number of genes in that pathway. *P.adjust* means the *P* values of GO and KEGG analysis were adjusted by false discovery rate. **E** Fractional representation of 22 immune cell types in both MVI-positive and MVI-negative samples. **F** Boxplot illustrating the differences in immune-infiltrating cells between MVI-positive and MVI-negative samples. Statistical significance is denoted by *p < 0.05. DEGs, differentially expressed genes; GO, gene ontology; KEGG, Kyoto Encyclopedia of Genes and Genomes; MVI, microvascular invasion; NK, natural killer; PPI, protein–protein interaction; STRING, search tool for retrieval of interacting genes/proteins

Gene ontology (GO) enrichment analysis revealed that in the biological processes category, MVI-related genes were primarily involved in metabolic process and regulation of synapse (Fig. 4A, C). Kyoto Encyclopedia of Genes and Genomes (KEGG) enrichment analysis further demonstrated that these DEGs were mainly enriched in pathways such as the Wnt signaling pathway, Neuroactive ligand-receptor interaction, and Hippo signaling pathway (Fig. 4D).

To explore the functional interactions of these DEGs, we utilized the search tool for retrieval of interacting genes/proteins (STRING) database to construct a protein–protein interaction (PPI) network and identified the top ten hub genes that played central roles in the network (Fig. 4B), primarily involved in ion channel activity, signal transduction, and the epithelial-mesenchymal transition (EMT) process.

In addition, we employed the CIBERSORT algorithm to analyze the immune cell infiltration associated with MVI in HCC patients. The distribution of 22 immune cell types across the samples is illustrated in Fig. 4E. Compared to MVI-negative HCC patients, MVI-positive HCC patients exhibited significantly higher infiltration levels of regulatory T cells (Tregs) and M0 macrophages, whereas monocyte and M2 macrophage infiltration levels were notably reduced (Fig. 4F).

## Discussion

This retrospective multi-center study aimed to develop and validate CR, SE-DL, and AD-DL models for the non-invasive preoperative prediction of MVI status in HCC using clinical and dynamic contrast-enhanced magnetic resonance imaging features from 546 patients across five centers and the TCIA cohort. The AD-DL model trained with IP, OP, and AP sequences demonstrated the highest diagnostic performance and generalizability. This suggests that patients identified as high-risk for MVI by the AD-DL model based on the IP and OP AP images may require a wider surgical margin; for HCCs ≤ 3 cm with a predicted high risk of MVI, liver resection may be preferable to radiofrequency ablation to reduce the risk of recurrence [11–13]. Additionally, MVI predictions using this model successfully stratified HCC patients for ERFS (*p* < 0.05). DEGs associated with the MVI status predicted by the AD-DL model, based on the optimal sequence combination of IP, OP, and AP, were primarily enriched in the metabolic processes and the Wnt signaling pathway, and associated with specific infiltrative immune cell types, thereby increasing the model’s interpretability and promoting its broader clinical application.

MRI-based deep learning models have been extensively investigated for predicting MVI status; however, multi-center studies are limited [33], and the generalizability of these models across different centers remains uncertain, which impedes their wider adoption. A multi-center study by Wang et al [33] developed an MRI-based deep learning model, which achieved a highest AUC of 0.612 (95% CI: 0.436–0.732) on the external test set for single-task models. This AUC was significantly lower than that of our AD-DL model trained with IP, OP, and AP sequences on the external test set (*Z* test, *p* < 0.05). One possible explanation for this suboptimal performance is that the training data were derived from a single-center hospital data, and differences in MRI acquisition parameters among institutions—such as image resolution, signal-to-noise ratio, slice thickness, and image quality—introduced data biases, limiting the model’s generalizability. In the study by Wang et al [33], the multi-task model showed a significant improvement in predictive performance compared to the single-task model, achieving a highest AUC of 0.837 (95% CI: 0.778–0.893) in the external test cohort. The AUC, sensitivity, specificity, and ACC of their multi-task model showed no significant difference compared to our AD-DL model (Table S5). However, as multi-task models are typically composed of both shared and task-specific subnetworks, their increased architectural complexity may potentially affect the interpretability of predictions for individual tasks [34].

Training models using data from multiple source domains (hospitals) and validating them on unseen target domains (i.e., multiple external test sets) is an effective approach to achieve domain generalization [21, 22]. Furthermore, extracting domain-invariant deep features using adversarial networks can enhance model generalizability, ensuring that the extracted features are both discriminative for MVI and invariant to biases across different MRI datasets. In this study, we trained models using data from three hospitals and employed adversarial networks to learn domain-invariant deep features. Considering the potential biases in liver MRI sequences across hospitals and the need for simple yet robust models for better generalizability, we compared the diagnostic performance of deep learning models based on different combinations of MRI sequences. The optimal model was the AD-DL model trained with IP, OP, and AP sequences, which outperformed the SE-DL and CR models based on the same sequences in two independent external test sets in terms of AUC, ACC, and sensitivity, without compromising specificity. Previous studies have utilized T2WI, PVP, and DP in MRI to predict MVI [19, 20, 33]. In our study, however, the combination of T2WI, PVP, and DP yielded an AUC of less than 0.7 in the test set. This may be attributed to several factors. T2WI is more susceptible to artifacts due to its inherent sequence properties and physiological motion in the abdomen [35]. Additionally, variations in acquisition parameters and timing of PVP and DP across different centers may affect the capture of tumor and peritumoral enhancement characteristics at the microstructural level. These inconsistencies could impair the generalizability of deep learning models for MVI prediction across multiple centers. Given that most HCCs exhibit heterogeneous AP hyperenhancement, which reflects tumor heterogeneity [28], it is inferred that AP images may contain more information relevant to MVI. IP/OP imaging provides crucial information regarding fat and iron content [36], both of which are important in differentiating HCC [28]. Although previous studies and our findings showed no direct correlation between fat or iron content as assessed by MRI and MVI [29–32], these analyses were based on macroscopic findings visible to the human eye, which lack the depth of microscopic detail. Deep learning may extract quantitative microscopic features from IP/OP images that are closely associated with MVI. This aspect has been overlooked in prior bi- or multi-center MRI-based deep learning studies for MVI, warranting further investigation.

Deep learning models are often considered “opaque”. Enhancing model interpretability is crucial for improving clinicians’ trust and acceptance, ensuring diagnostic safety and ACC, and promoting the broader clinical adoption of these models [37]. Some previous studies [17, 18, 33] used gradient-weighted class-activation (Grad-CAM) to enhance the interpretability of MRI-based deep learning models for MVI to a certain extent. In this study, we also employed Grad-CAM, revealing that the heatmaps of MVI-positive HCCs were primarily concentrated in the tumor margins, consistent with previous findings that over 85% of MVI occurs in peritumoral regions [7]. However, Grad-CAM primarily highlights localized regions related to the target prediction, which makes it challenging to integrate with broader prior medical knowledge, thereby limiting clinician acceptance. This study utilized biological function analysis, identifying that the hub gene Bmp4, associated with MVI status, was related to the migration efficiency of microvascular endothelial cells [38]. Furthermore, DEGs related to MVI were predominantly enriched in processes such as EMT and the Wnt signaling pathway. EMT is a primary mechanism for metastasis in MVI-positive HCC and is activated by Wnt/β-catenin signaling, which promotes HCC invasion and metastasis [39, 40]. Regarding immune cell infiltration in the tumor microenvironment, we observed increased levels of Tregs in MVI-positive HCC patients. Tregs have been shown to promote HCC growth, progression, and resistance to sorafenib [41], which may partially explain the poor response to immunotherapy in MVI-positive HCC patients. By linking the imaging-based deep learning model for MVI prediction with genomic alterations through biological function analysis, this study reflects the biological relevance of the model at the genetic and mechanistic levels, further enhancing interpretability and facilitating clinical translation.

Our study has several limitations. First, the retrospective nature of the study may introduce inherent biases. Second, the dataset predominantly consisted of East Asian patients with HBV-related HCC, necessitating further validation of the model in diverse patient populations. Third, the associations between imaging features and transcriptomics are preliminary, and the results may be influenced by the small sample size. To improve the reliability of these findings, future studies should include larger, well-designed, global multi-center prospective datasets for model validation and generalization.

In conclusion, the MRI-based AD-DL model developed in this study can predict MVI status in HCC patients, stratify prognosis, and demonstrate high generalizability. In addition to using Grad-CAM to identify and highlight key regions in MRI images, we also incorporated bioinformatic analyses of DEGs related to MVI. This approach further enhanced the model’s interpretability and facilitated its clinical translation. Nevertheless, these findings require prospective validation in randomized trials to assess the clinical applicability of our imaging model alongside clinical standards and promote personalized treatment strategies.

## Supplementary information

Electronic Supplementary Material

## Data Availability

The data analyzed and the codes used during the current study are available from the corresponding author on reasonable request.

## Abbreviations

ACC: Accuracy
AD-DL: Adversarial network-deep learning
AFP: Alpha-fetoprotein
AIC: Akaike information criterion
AP: Arterial phase
AUC: Area under curve
CI: Confidence interval
CR: Clinical-radiological
DEGs: Differentially expressed genes
DP: Delayed phase
EMT: Epithelial-mesenchymal transition
ERFS: Early recurrence-free survival
GO: Gene ontology
Grad-CAM: Gradient-weighted class-activation
HBV: Hepatitis B virus
HCC: Hepatocellular carcinoma
IP: In-phase
KEGG: Kyoto Encyclopedia of Genes and Genomes
MVI: Microvascular invasion
OP: Opposed-phase
OR: Odds ratio
PPI: Protein–protein interaction
PVP: Portal venous phase
ROC: Receiver operating characteristic
SE-DL: Squeeze-and-excitation-deep learning
SENet: Squeeze-and-excitation network
T2WI: T2-weighted imaging
TCIA: The cancer imaging archive
Tregs: Regulatory T cells
