# Supplementary material for: Interpretable and generalizable deep learning model for preoperative assessment of microvascular invasion and outcome in hepatocellular carcinoma based on MRI: a multicenter study
Source: Insights Imaging. 2025 Jul 3;16:151. doi: 10.1186/s13244-025-02035-0 (PMC12229396; doi:10.1186/s13244-025-02035-0)
Supplement: Supplementary file 1 — Electronic Supplementary Material [file 13244_2025_2035_MOESM1_ESM.pdf]

Interpretable and generalizable deep learning model for preoperative assessment of microvascular invasion and outcome in hepatocellular carcinoma based on MRI: a multicenter study

ELECTRONIC SUPPLEMENTARY MATERIAL

Supplementary Appendix
Supplementary References
Supplementary Tables

Supplementary Appendix 1:

Inclusion and Exclusion Criteria

The inclusion criteria were as follows: (a) patients with pathology diagnosis of HCC; (b) patients underwent extracellular contrast medium-magnetic resonance imaging (ECA-MRI) within one month before hepatectomy; (c) patients with MVI documentations; and (d) patients without prior treatment for HCC. The exclusion criteria included: (a) incomplete MRI data; and (b) image artifacts.

Clinical data and outcomes

Collect the following clinical data: age, sex, hepatitis B virus (HBV) infection status, Barcelona Clinic Liver Cancer (BCLC) stage, Child-Pugh classification, levels of alanine aminotransferase (ALT), aspartate aminotransferase (AST), plasma albumin (ALB), prothrombin time (PT), and alpha-fetoprotein (AFP).

All patients at the Taizhou center were followed up every 3 to 6 months after liver resection with AFP testing and imaging examinations to monitor for disease progression, including local recurrence or distant metastasis. The final follow-up date for this study was January 31, 2022. For patients lost to follow-up, survival time was defined as the interval from surgery to the last documented follow-up.

MVI Evaluation

Evaluation was performed on surgically resected specimens using the "7-point" baseline sampling method. Each specimen was independently assessed by two pathologists from the respective centers, with any discrepancies resolved through discussion to reach consensus. To ensure accuracy and consistency in MVI evaluation, all participating pathologists underwent standardized training prior to the assessment.

MRI Scan

For dynamic imaging, after a bolus injection of 0.1 mmol/kg body weight of Gadopentetate Dimeglumine (Gd-DTPA, Magnevist, Bayer Schering, Berlin, Germany), images were obtained in the hepatic arterial phases (20 seconds), portal venous phases (60 seconds), and delayed phases (180 seconds).

MRI features

All features were retrospectively assessed by two radiologists (M.C.L. and D.X, with 15 and 8 years of expertise in abdominal imaging diagnosis, respectively), and were blinded to the clinical and pathological data. In cases of disagreement between the two radiologists, a third, more senior radiologist (Y.D.W, with 21 years of experience in abdominal imaging diagnosis), was consulted. If a patient had multiple tumor lesions, the largest lesion was evaluated.

The following 14 imaging features were assessed: (1) maximum tumor diameter: the largest outer-edge-to-outer-edge dimension of the main lesion[1]; (2) tumer number[2]; (3) hypo-intensity on T1WI: lower lesion signal intensity than background on T1WI[2]; (4) blood products in mass: intralesional or perilesional hemorrhage in the absence of biopsy, trauma or intervention; a hyperintense area on T1-weighted images, with variable signal intensity on T2-weighted images[1];(5) rim arterial phase hyperenhancement(rim-APHE): spatially defined subtype of APHE in which arterial phase enhancement is most pronounced in observation periphery[1];(6) intratumor vascularity: which is the persistence of discrete arterial enhancement within the tumor in the arterial phase[3];(7) intratumor necrosis: a hypoattenuated central area on the nonenhanced images without enhancement during the postcontrast phases;(8) enhancing "capsule": smooth, uniform, sharp border around most or all of an observation, unequivocally thicker or more conspicuous than fibrotic tissue around background nodules, and visible as as enhancing rim in portal venous phase(PVP), delayed phase(DP); (9) incomplete "capsule": an absence of "capsule" (regardless of enhancement) or presence of disrupted "capsule" in any imaging plane[4];(10) nonperipheral washout: nonperipheral visually assessed temporal reduction in enhancement in whole or in part relative to composite liver tissue from earlier to later phase resulting in hypoenhancement in the extracellular phase[1];(11) nonsmooth tumor margin: nonnodular tumors or nodular tumors with irregular margin and a budding portion at the tumor periphery in any imaging plane[4,5]; (12) peritumoral mild-to-moderate T2 hyperintensity: the presence of wedge-shaped or flame-like area adjacent to the tumor with T2-weighted imaging of which the signal intensity is mildly or

moderately higher than that of liver and similar to or less than that of non-iron-overloaded spleen[4]; (13) peritumoral PVP hypoenhancement: the presence of wedgeshaped or flame-like hypoenhancing area adjacent to the tumor on PVP images[4]; (14) peritumoral arterial phase hyperenhancement: a detectable portion of crescent-shaped or polygonal enhancement outside the tumor margin with broad contact with the tumor border in arterial phase, becoming isointense with background liver parenchyma in the delayed phase[5].

Supplementary Appendix 2:

Proposed deep learning network architecture

Preprocessing:

The tumor regions were manually delineated by two experienced radiologists (D.X. and Z.J.X., with 8 and 5 years of expertise in abdominal imaging, respectively) on the maximum cross-sectional tumor areas of seven sequences: in-phase (IP) and opposed-phase (OP), T2-weighted imaging (T2WI), and non-contrast phase (NCP), the arterial phase (AP), portal venous phase (PVP), and delayed phase (DP). This was subsequently confirmed by a senior radiologist with 15 years of experience in abdominal radiology (Y.D.W.). The lesion areas were then cropped and subjected to gray-level normalization using specific coding. The cropped images from different sequences were resized to 64×64 pixels and concatenated along the channel dimension to create multi-sequence fusion data. Through different channel combinations within the convolutional neural network, experiments were conducted using various sequence combinations for further analysis.

For each patient, among the seven imaging sequences, we identified the 2D slice containing the largest lesion area within the HCC region annotated by the radiologist, and then cropped and standardized the lesion area. This method allowed us to extract the region of interest (ROI) for each sequence, which was subsequently resized to 64×64 pixels. Multiple ROIs were then concatenated along the channel dimension to generate multi-sequence fused data. The fused datasets

could be formed from various sequence combinations. For example, a multi-sequence dataset may consist of IN, OUT, and AP, resulting in a data shape of 64×64×3, or it may include T2WI, AP, PVP, and DP, yielding a data shape of 64×64×4. By inputting different sequence combinations into the model for training, we obtained experimental results corresponding to each specific combination.

Network:

Our network architecture consists of four components: a feature extraction module, an MVI classifier, a hospital classifier, and a gradient reversal layer. We employed squeeze-and-excitation network (SENet) without its classifier as the feature extractor, which mainly consists of eight squeeze-and-excitation (SE) Blocks. Each SE Block is formed by adding an SE Layer to the residual blocks of ResNet-18. After processing through the feature extraction module and flattening, the resulting feature vector has a length of 512. The MVI classifier is a linear layer with an input of 512 and an output of 2, designed to classify the presence or absence of MVI. The hospital classifier is another linear layer with an input of 512 and an output of 3, which is used to identify the hospital source of the multi-sequence fusion data. The hospital classifier functions as a domain classifier, where identifying the originating hospital for the multi-sequence fusion data is equivalent to determining the data's source domain. The hospital classifier connects to the feature extraction module via a gradient reversal layer. The gradient reversal layer performs an identity transformation during forward propagation. In backpropagation, it multiplies the gradient by a negative scalar λ, effectively reversing the gradient direction.

To suppress noise signals from the domain classifier in the early training stages, we do not use a fixed λ. Instead, λ is gradually increased from 0 to 1 as training progresses, specifically:

$$\lambda = \frac{2}{1 + \exp\left(-10 \cdot p\right)} - 1$$

where p represents the percentage of the training process completed.

The presence of the gradient reversal layer creates an adversarial relationship between the feature extraction module and the hospital classifier: the feature

extraction module strives to extract domain-invariant features, independent of any specific hospital (domain), to enable the MVI classifier to make accurate predictions. Conversely, the hospital classifier aims to correctly identify the source hospital of the data, relying on features that are indicative of hospital (domain) classification. During training, the feature extraction module progressively learns to extract domain-invariant features that are unrelated to specific hospitals (domains), while the hospital classifier becomes increasingly incapable of distinguishing the source hospital of the training data.

Our network mainly designed two loss functions: the L_m for the MVI classifier, and the L_d for the hospital (domain) classifier. L_m uses *Focal Loss* due to the imbalance in the training dataset, where negative samples far outweigh positive samples. The formula for L_m is as follows:

$$L_m = -\alpha(1 - p_t)^{\gamma} log(p_t)$$

Where p_t is the prediction probability of the model on the target class. α is the balance factor, which is used to adjust the influence weight between the negative and positive samples. γ is the focusing factor, which is used to adjust the weight of the difficult and easy samples. During the training process, α is taken as 0.75 for positive samples, α is taken as 0.25 for negative samples, and γ is taken as 3. Such values are used to weigh the influences between positive samples and negative samples, and between difficult samples and easy samples. L_d is the cross-entropy loss function. The total loss function is shown below:

$$L_{total} = L_m + \beta \cdot L_d$$

Where β is used to balance the influence of the two loss functions, and 0.03 is taken in the training process.

Supplementary Appendix 3:

Analysis of the Biologic Function

There were 40 HCC patients in the TCIA database (TCGA-LIHC) with preoperative MRI. Of which, 3 patients were excluded due to prior treatments, 5 due to incomplete MRI data, and 5 due to image artifacts. A total of 27 patients with complete MRI images and RNA-sequence data were included.

RNA was extracted from tumor tissue samples. All cells from the tumor specimens were used for immune cell identification at the bulk RNA-seq level. Gene expression levels were calculated using Fragments Per Kilobase of transcript per Million mapped reads (FPKM) formula, and differentially expressed genes (DEGs) were identified using Count formula. The “DESeq” package was employed to analyze DEGs associated with MVI, with a threshold set at an adjusted p-value < 0.05. DEGs related to MVI were identified based on the arterial phase, in-phase and out-phase sequence combinations, and adversarial training in deep learning. MVI-related DEGs are defined as DEGs obtained based on image features.

For the total of 198 MVI-related DEGs, Gene Ontology (GO) and Kyoto Encyclopedia of Genes and Genomes (KEGG) functional enrichment analyses were performed using the “clusterProfiler” package, with thresholds set at p-value < 0.05 and q-value < 0.05. Additionally, a protein-protein interaction (PPI) network for DEGs was constructed using the Search Tool for Retrieval of Interacting Genes/Proteins (String) database (https://string-db.org/), and the top 10 hub genes were identified using the maximal clique centrality (MCC) algorithm in Cytoscape software (Version 3.9.0). The “CIBERSORT” package was used to assess the types and quantities of immune cell infiltration in MVI-negative and MVI-positive HCC samples. Differences in immune cell infiltration were evaluated using the Wilcoxon test, with a significance threshold of $p < 0.05$.

Supplementary References

1 Chernyak V, Fowler KJ, Kamaya A, et al. Liver Imaging Reporting and Data System (LI-RADS) Version 2018: Imaging of Hepatocellular Carcinoma in At-Risk Patients. Radiology. 2018;289:816-830. https://doi.org/10.1148/radiol.2018181494.

2 Hong SB, Choi SH, Kim SY, et al. MRI Features for Predicting Microvascular Invasion of Hepatocellular Carcinoma: A Systematic Review and Meta-Analysis. Liver Cancer. 2021;10:94-106. https://doi.org/10.1159/000513704.

3 Yang J, Dong X, Wang G, et al. Preoperative MRI features for characterization of vessels encapsulating tumor clusters and microvascular invasion in hepatocellular carcinoma. Abdom Radiol (NY). 2023;48:554-566. https://doi.org/10.1007/s00261-022-03740-w.

4 Jiang H, Wei H, Yang T, et al. VICT2 Trait: Prognostic Alternative to Peritumoral Hepatobiliary Phase Hypointensity in HCC. Radiology. 2023;307:e221835. https://doi.org/10.1148/radiol.221835.

5 Min JH, Lee MW, Park HS, et al. Interobserver Variability and Diagnostic Performance of Gadoxetic Acid-enhanced MRI for Predicting Microvascular Invasion in Hepatocellular Carcinoma. Radiology. 2020;297:573-581. https://doi.org/10.1148/radiol.2020201940.

Supplementary Tables

Table S1. The MRI image acquisition parameters of the multiple centers.

Hospital	Scanner	Sequence	TR/TE(ms)	FOV(mm^2)	Matrix	Slice Thickness(mm)	Flip Angle(°)
Beijing Friendship Hospital, Capital Medical University (Beijing center)	GE 3.0T (Discovery)	T1WI	4.12/1.936	360×360	256×224	4	12
		T2WI	7500/87.372	400×400	320×224	6	111
	GE 1.5T (Signa)	T1WI	3.28/1.568	380×380	256×160	4.4	15
		T2WI	7500/86.02	380×380	288×224	6	90
	Siemens 3.0T (Prisma)	T1WI	3.76/1.23	360×360	256×224	3	10
		T2WI	4392.9375/83	400×400	320×224	6	72
Sir Run Run Shaw Hospital, School of Medicine, Zhejiang University (Hangzhou center)	Siemens 3.0T (Skyra)	T1WI	3.45/1.32	380×380	320×260	3	12
		T2WI	4434.6875/83	380×380	320×320	6	112
	GE 3.0T (Signa)	T1WI	2.588/1.188	420×420	320×260	4.4	11
		T2WI	6315.79/87.156	380×304	320×224	8	90
	GE 3.0T (Discovery)	T1WI	4.425/1.78	420×420	320×260	4.4	12
		T2WI	7500/97.088	380×304	320×224	7	142
Ningbo Hwamei Hospital, University of Chinese Academy of Sciences (Ningbo center)	Siemens 3.0T (Prisma)	T1WI	4/1.31	360×300	266×222	3	9
		T2WI	2500/75	380×300	140×154	6.5	160
	Siemens 1.5T (Avanto)	T1WI	3.49/1.02	350×260	233×173	4	10
		T2WI	1200/98	350×300	260×222	7	160
	Philips 1.5T (Ingenia)	T1WI	4/1.9	350×260	233×173	6	15
		T2WI	2127.3037109375/80	350×300	260×222	6	90
Taizhou Hospital, Zhejiang University (Taizhou center)	GE 3.0T (Discovery)	T1WI	3.692/1.674	324×360	260×224	5	12
		T2WI	13333.333/76.42687	400×400	320×320	6	110
	GE 1.5T (Signa)	T1WI	3.768/1.82	315×350	288×170	5	12
		T2WI	7058.82/88.596	400×300	228×224	8	90
The Second Affiliated Hospital of Dalian Medical University (Dalian center)	Siemens 3.0T (Verio)s	T1WI	3.92/1.39	360×360	256×224	3	9
		T2WI	3914.0909090909/105	400×400	320×224	7	140
	GE 1.5T (Signa)	T1WI	6.132/3.126	320×350	280×170	6	15
		T2WI	12857.1/92.276	400×300	228×224	6	90

Table S2. The Clinical and Radiological Characteristics of patients in each set.

Characteristic	[a]Training Set (n=263)	[b]Internal Test Set (n = 66)	[c]External Test Set1 (n = 93)	[d]External Test Set2 (n = 97)	*P* value
**Patient demographics**					
Age (>50 years)	202(76.8)	55(83.3)	79(84.9)	87(89.7)	0.029[ad]
Sex (male)	211(80.2)	56(84.8)	71(76.3)	75(77.3)	0.552
HBV infection	217(82.5)	52(78.8)	88(94.6)	76(78.4)	0.010[cd,bc,ac]
BCLC stage (0 or A)	241(91.6)	64(97)	77(82.8)	92(94.8)	0.005 [bc,ac]
Child-Pugh grade (A) (1/2)	202(76.8)	45(68.2)	77(82.8)	50(51.5)	<0.001[ad,bd,cd,bc]
ALT level (>50 U/L)	67(25.5)	19(28.8)	13(14.0)	64(66.0)	<0.001[cd,ad]
AST level (>40 U/L)	87(33.1)	23(34.8)	30(32.3)	45(46.4)	0.106
ALB level (>40 g/L) (1/2)	145(55.1)	36(54.5)	59(63.4)	29(29.9)	<0.001 [ad,bd,cd,]
PT level (>13s)	153(58.2)	44(66.7)	70(75.3)	77(79.4)	<0.001[ad,ac]
AFP level (>200 ng/mL)	72(27.4)	14(21.1)	25(26.9)	25(25.8)	0.785
**Radiologic feature**					
Maximum tumor diameter (>5 cm)	89(33.8)	21(31.8)	18(19.4)	29(29.9)	0.074
Tumer number (solitary)	242(92)	63(95.5)	85(91.4)	89(91.8)	0.777
Hypo-intensity on T1WI (present)	227(86.3)	58(87.9)	82(88.2)	81(83.5)	0.788
Blood products in mass (present)	67(25.5)	13(19.7)	16(17.2)	27(27.8)	0.248
Rim APHE (present)	12(4.6)	2(3)	3(3.2)	5(5.2)	0.864
Intratumor vascularity (present)	132(50.2)	29(43.9)	46(49.5)	35(36.1)	0.104
Intratumor necrosis (present)	54(20.5)	11(16.7)	11(11.8)	14(14.4)	0.218
Enhancing capsule (present)	240(91.3)	58(87.9)	82(88.2)	85(87.6)	0.662
Incomplete “capsule” (present)	210(79.8)	59(89.4)	76(81.7)	70(72.2)	0.057
Nonperipheral washout (present)	224(85.2)	53(80.3)	72(77.4)	73(75.3)	0.117
Nonsmooth tumor margin (present)	181(68.8)	46(69.7)	61(65.6)	63(64.9)	0.851
Peritumoral mild-to-moderate T2 hyperintensity (present)	44(16.7)	6(9.1)	10(10.8)	13(13.4)	0.098
Peritumoral PVP hypoenhancement (present)	55(20.9)	10(15.2)	14(15.1)	10(10.3)	0.287
peritumoral AP hyperenhancement (present)	77(29.3)	19(28.8)	26(28)	18(18.6)	0.226
**MVI (present)**	100(38.0)	26(39.4)	27(29.0)	35(36.1)	0.434

a Training Set; b Internal Test Set; [c] External Test Set1; d External Test Set2; ac, ad, bc, bd and cd, the *p* value are significant between the sets.

Abbreviations: AFP, alpha-fetoprotein; ALB, plasma albumin; ALT, alanine aminotransferase; AP, arterial phase; APHE, arterial phase hyperenhancement; AST, aspartate aminotransferase; HBV, hepatitis B virus; BCLC, Barcelona Clinic Liver Cancer; PT, prothrombin time; PVP, portal venous phase; MVI, microvascular invasion.

Table S3. Interobserver Agreement of MR Features of HCC.

Radiologic feature	κ value
Maximum tumor diameter (cm)*	0.936
Tumer number (solitary)	0.943
Hypo-intensity on T1WI	0.911
Blood products in mass	0.924
Rim APHE	0.801
Intratumor vascularity	0.878
Intratumor necrosis	0.832
Enhancing capsule	0.847
Incomplete “capsule”	0.809
Nonperipheral washout	0.715
Nonsmooth tumor margin	0.849
Peritumoral mild-to-moderate T2 hyperintensity	0.847
Peritumoral PVP hypoenhancement	0.834
Arterial peri-tumoral enhancement	0.917

Abbreviations: APHE, arterial phase hyperenhancement; HCC, hepatocellular carcinoma; MR, magnetic resonance; PVP, portal venous phase.

Table S4. Diagnostic efficacy of SE-DL model and AD-DL model for sequence combinations with AUC > 0.7

Sequence	AD-DL Internal Test Set	SE-DL Internal Test Set	AD-DL External Test Set1	SE-DL External Test Set1	AD-DL External Test Set2	SE-DL External Test Set2
OP, AP, PVP	0.753	0.725	0.688	0.697	0.692	0.622
IP, OP, T2WI, PVP	0.746	0.707	0.715	0.645	0.651	0.680
IP, T2WI, AP, PVP	0.654	0.704	0.604	0.621	0.625	0.626
OP, T2WI, NCP	0.736	0.734	0.566	0.589	0.709	0.708
IP, OP, T2WI, NCP, AP	0.716	0.703	0.704	0.694	0.660	0.666
T2WI, NCP, AP	0.725	0.763	0.631	0.630	0.652	0.683
IP, OP, PVP	0.799	0.766	0.639	0.692	0.687	0.671
OP, NCP, AP	0.716	0.713	0.666	0.675	0.670	0.654
IP, AP, PVP	0.730	0.732	0.708	0.683	0.665	0.623
OP, NCP, PVP	0.738	0.731	0.709	0.700	0.703	0.652
IP, OP, DP	0.768	0.746	0.723	0.709	0.762	0.724
IP, OP, NCP	0.735	0.739	0.700	0.703	0.690	0.671
IP, PVP	0.718	0.733	0.745	0.622	0.685	0.593
IP, OP, AP	0.793	0.807	0.801	0.762	0.773	0.696
T2WI, AP	0.710	0.701	0.632	0.686	0.699	0.709
OP, T2WI	0.754	0.707	0.608	0.640	0.608	0.654
IP, NCP, AP	0.733	0.718	0.682	0.653	0.625	0.688
OP	0.742	0.766	0.590	0.647	0.771	0.759
OP, PVP, DP	0.681	0.708	0.600	0.556	0.729	0.675
IP, T2WI, PVP, DP	0.702	0.701	0.602	0.572	0.588	0.609

Abbreviations: AD-DL, adversarial network-deep learning; AP, arterial phase; AUC, area under curve; DP, delayed phase; IP, in-phase; NCP, non-contrast phase; OP, opposed-phase; PVP, portal venous phase; SE-DL, squeeze-and-excitation-deep learning; T2WI, T2-weighted imaging.

Table S5. Comparison with Existing Literature

Index	*AD-DL Model (External Test1 Set1)	*[a]Multitask Model (External test set 1)	Z value	*P* Value
MVI+	27	82		
MVI-	66	130		
AUC (95% CI)	0.801 (0.706–0.877)	0.837 (0.778–0.893)	0.686	0.508
Sensitivity	0.630	0.816	1.82	0.069
Specificity	0.818	0.698	-1.93	0.053
Accuracy	0.763	0.744	-0.36	0.720

*The external test set with the highest AUC in study

[a]From reference 33 in the main manuscript.

Abbreviations: AD-DL, adversarial network-deep learning; AUC, area under curve; MVI, microvascular invasion.
